# Transactions of the Indiana State Medical Society, at Its Eleventh Annual Session, Held in Indianapolis, May 17th and 18th, 1860

**Published:** 1860-12

**Authors:** 


Transactions of the Indiana State Medical Society, *at its Eleventh Annual Session, held in Indianapolis, May* 17*th and* 18*th*, 1860

This is a Pamphlet of 68 pages, the record of Proceedings of the Annual Meeting; the Presidential Address, by Dr. David Hutchinson; a Paper on Artificial Lactation, by Dr. Charles M. Wetherill; on Medical Inhalation, by Dr. T. W. Fry; on the Progress of Medicine, by Dr. J. H. Brower; on Diphtheria, by Dr. R. E. Haughton; on Medical Education, by Dr. Charles Fishback; also the Constitution and By-Laws of the Society, and a List of Members. Nearly all of these Papers possess the merit of brevity, and may be read with profit. The following are the conclusions of Dr. Haughton in relation to Diphtheria:

"*Summary.*—1. Diphtheria is a specific disease, as is seen in its history, its progress, its mode of extension, and more particularly in the character of its exudation; also in its choice of locality, in its toxic influences, its termination and its sequelæ.

"2. It is often confounded with scaralatina angina, with cynanche gangrenosum, with ulcerative tonsillitis, and may be, even, with cynanche trachealis.

"3. It is not fully determined to be contagious or infectious. It is both epidemic and endemic, and prevails with much severity in limited centers of population.

"4. It presents itself under three varieties:—I. Simple diphtheria. II. Laryngeal or croupal diphtheria. III. Malignant. The first is easily managed; the second is very dangerous; the third nearly always fatal.

"5. The simple variety may be treated with mild stimulating gargles; the other varieties energetically with the internal administration of quinine, pot. chlor., mineral acids and vegetable tonics. The tr. ferri chlor. both internally and locally; a strong solution of the argent. nit. to the throat, and a solution of chloride sodium, as a disinfectant. As adjuvants: ventilation, nutritious diet, stimulants and sponging.

"6. Tracheotomy is contra-indicated in all cases."

